# Eight weeks of creatine monohydrate supplementation is associated with increased muscle strength and size in Alzheimer’s disease: data from a single-arm pilot study

**DOI:** 10.3389/fnut.2025.1670641

**Published:** 2025-09-04

**Authors:** Aaron N. Smith, Debra K. Sullivan, Jill K. Morris, Aaron F. Carbuhn, Trent J. Herda, Matthew K. Taylor

**Affiliations:** ^1^Department of Dietetics and Nutrition, University of Kansas Medical Center, Kansas City, KS, United States; ^2^Alzheimer’s Disease Research Center, University of Kansas, Fairway, KS, United States; ^3^Kansas University Diabetes Institute, University of Kansas Medical Center, Kansas City, KS, United States; ^4^Kansas Center for Metabolism and Obesity Research, University of Kansas Medical Center, Kansas City, KS, United States; ^5^Department of Neurology, University of Kansas Medical Center, Kansas City, KS, United States; ^6^Department of Health, Sport, and Exercise Sciences, University of Kansas, Lawrence, KS, United States

**Keywords:** Alzheimer’s disease, creatine monohydrate, muscle strength, non-pharmacological intervention, muscle cross-sectional area, neuromuscular junction

## Abstract

**Objective:**

To investigate the potential muscular benefits of an eight-week creatine monohydrate (CrM) supplementation in patients with Alzheimer’s disease (AD).

**Methods:**

This single-arm pilot trial, conducted at the University of Kansas Medical Center in Kansas City, examined the intervention-associated changes in muscle strength, muscle size, and neuromuscular junction (NMJ) integrity following 8 weeks of CrM supplementation (20 g/day) in 20 participants with AD. All participants completed handgrip-strength measurements on the dominant hand (highest of three trials in kg of force). Ten participants completed lower body strength assessment via leg dynamometry at three velocities (1.05 rad∙s^−1^, 2.10 rad∙s^−1^, 3.14 rad∙s^−1^), with peak torque (in Newton-meters) recorded over five repetitions. Eighteen participants completed muscle size assessment by ultrasound measurement of cross-sectional area (mCSA, cm^2^) in the rectus femoris and vastus medialis, as well as muscle thickness (cm) in the rectus femoris, vastus medialis, and vastus lateralis. NMJ integrity was assessed in 19 participants by measuring plasma C-terminal agrin fragment (CAF) levels. All assessments were measured at baseline and 8 weeks.

**Results:**

Following 8 weeks of CrM, mean hand-grip strength increased by 1.9 kg from baseline (*p* = 0.02). Lower leg strength did not change for any velocity among the ten participants who completed leg dynamometry. mCSA (*n* = 18) increased from baseline in the rectus femoris (*p* = 0.03) and vastus medialis (*p* = 0.01), but muscle thickness (*n* = 18) did not change in the rectus femoris (*p* = 0.41), vastus medialis (*p* = 0.37), nor vastus lateralis (*p* = 0.17). Subcutaneous fat (*n* = 18) decreased in the rectus femoris region (*p* = 0.006) and vastus lateralis region (*p* = 0.003), with no change in the vastus medialis region (*p* = 0.52). Mean CAF (*n* = 19) values did not change (*p* = 0.46).

**Conclusion:**

This eight-week pilot trial suggests that 20 g/day of CrM may provide modest skeletal muscle benefits in patients with AD. These data provide preliminary evidence to warrant further investigation of the potential for CrM to prevent AD-related decline in muscle function.

**Clinical trial registration:**

ClinicalTrials.gov, identifier NCT05383833.

## 1 Introduction

The loss of physical function is common in Alzheimer’s disease (AD) and often presents as diminished muscle mass and strength (1). Changes in muscle strength and size appears to be not only a consequence of AD, limiting mobility and promoting frailty, but also a contributor to its risk and progression (1, 2). In an AD mouse model, neuromuscular dysfunction emerged before cognitive impairment (3), indicating that skeletal muscle changes occur early in the disease process and may be an underappreciated therapeutic target.

For instance, in older adults with mild cognitive impairment due to AD, six months of resistance training increased muscle strength and integrity, improved cognition, and attenuated brain amyloid burden and atrophy (4). In AD mouse models, direct manipulation of skeletal muscle (5) and stimulation of anabolic pathways that drive hypertrophy (6) also improved cognition, further implicating muscle as a modifiable node in the disease cascade. Thus, interventions that enhance muscle and strength may not only slow functional decline in AD but may also have broader disease-modifying potential, as suggested by preclinical studies (5, 6).

Creatine monohydrate (CrM) has substantial evidence for enhancing muscle strength and size (7–14), and our recent pilot trial suggests CrM may be associated with benefits in AD patients (7). The basis for these effects lies in creatine’s (Cr) role as an organic compound found primarily in skeletal muscle (15) that stores high-energy phosphates in the form of phosphocreatine (PCr) and helps maintain intracellular energy flux (16, 17). CrM supplementation increases intramuscular creatine stores, thereby expanding the capacity for PCr formation and ATP regeneration during high-intensity muscle contractions. This enhanced energy availability can support greater force production and facilitate the energy-demanding processes of muscle protein synthesis, potentially leading to improvements in muscle strength and size (8–13). In older adults, CrM supplementation has been shown to improve strength and function (12, 14); however, its effects on skeletal muscle have not been investigated in the context of AD.

The purpose of this single-arm pilot study was to investigate our hypothesis that 8 weeks of CrM supplementation improves muscle strength, size, and neuromuscular junction integrity (NMJ) in AD.

## 2 Materials and methods

### 2.1 Creatine to augment bioenergetics in Alzheimer’s study and participants

The Creatine to Augment Bioenergetics in Alzheimer’s study (7, 18) allocated 20 participants with a clinical diagnosis of probable AD-dementia (19) to the 20 g/day CrM intervention. As the primary outcome of the CABA trial was feasibility, a single-arm design was employed to assess tolerability, compliance, and preliminary efficacy signals to inform future randomized controlled trials. Participants were 60–90 years old, were on a stable dose of AD-related medications (e.g., donepezil or memantine) for at least 30 days, had a study partner, scored ≥17 on the Mini-Mental State Exam (MMSE) (20), spoke English as the primary language, and had the ability to perform leg strength exercises. Exclusion criteria included insulin-dependent diabetes, chemotherapy or radiation within the past 5 years, a recent cardiac event (e.g., myocardial infarction), diagnosis of another neurodegenerative disease, inability to undergo MRI, and participation in a clinical trial or investigational drug or therapy within 30 days of screening. Participants were encouraged to maintain regular dietary intake and physical activity levels during the study. The study protocol was approved by the University of Kansas Medical Center Institutional Review Board, and all participants provided informed consent in accordance with institutional guidelines.

### 2.2 CrM intervention

Participants consumed 20 g of powdered CrM (Life Extension Inc., United States) daily for 8 weeks, divided into two 10-gram doses, mixed into beverages of the participant’s choice. This two ×10 g dosing regimen was selected because it has been shown to be safe (21) and to minimize participant and study partner burden, as managing fewer daily doses would be easier for patients with AD and their study partners. To support adherence, research dietitians contacted the study partner weekly, and study partners completed a daily CrM tracker.

### 2.3 Physical-activity assessment

Baseline physical activity was measured with the two-item Stanford Brief Activity Survey (SBAS) (20), as physical activity may affect muscle strength and size. Study partners selected one statement that best described the participant’s usual on-the-job (or daily routine) activity and one that best described their leisure-time activity during the past year, each ranging from sedentary to vigorous. Responses were cross-referenced on the SBAS color-coded scoring table to classify each participant into one of five overall activity categories: inactive, light, moderate, hard, or very hard.

### 2.4 Muscle strength, size, and body composition acquisition

All muscular and body composition assessments were measured at baseline and 8 weeks.

#### 2.4.1 Handgrip strength

Handgrip strength was measured on the participant’s dominate hand using a calibrated Jamar hand dynamometer, a validated method for assessing upper body strength (22, 23). While seated in a chair with their feet flat on the floor, participants squeezed the dynamometer with maximal effort for at least 3 seconds per trial while the study team provided verbal encouragement. Each participant completed three sets, with at least 1 minute of rest between sets. The highest force value (kg) recorded across the three trials was used as the participant’s maximal handgrip strength.

#### 2.4.2 Leg muscle strength

A sub-sample of the last 10 participants to participate in CABA completed leg strength testing as described in detail by Herda et al. (24). Participants performed maximal isokinetic contractions of the right leg extensors using a calibrated Biodex isokinetic dynamometer (Biodex Corp., Shirley, NY), with the hip positioned at a 90° angle. Each participant completed five maximal contractions at three velocities (1.05 rad∙s^−1^, 2.10 rad∙s^−^1, 3.14 rad∙s^−1^), with 5 minutes of rest between each velocity. Study personnel provided verbal encouragement to elicit maximal effort and speed during each trial. Torque, position, and velocity signals were recorded using the Biodex system. Dynamometer signals were sampled at 2000 Hz, and torque data were low-pass filtered with a 10 Hz cutoff. Peak torque (Newton-meters; Nm) was calculated as the highest 0.25 s epoch from each contraction using custom-written software (LabVIEW 2019, National Instruments, Austin, TX). For each velocity, the highest peak torque value was used for analysis.

#### 2.4.3 Leg muscle morphology

**Figure 1:**
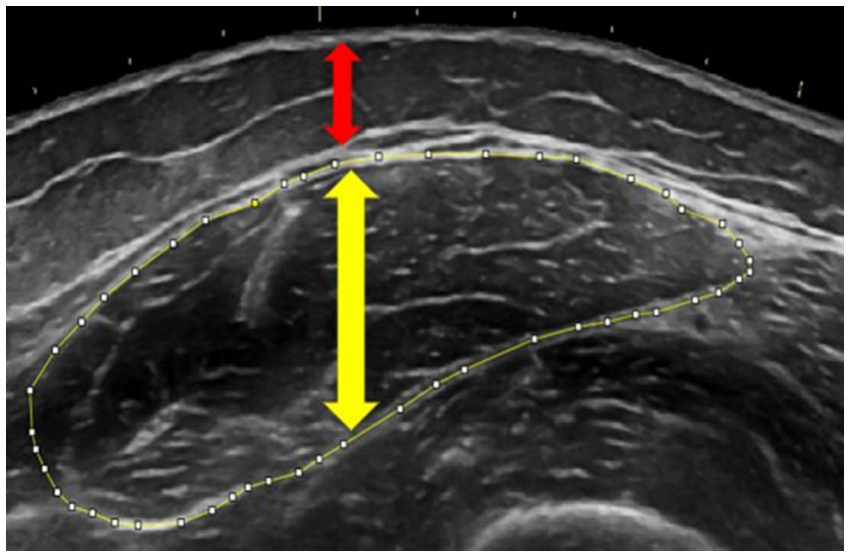
Ultrasonography image demonstrating measurements of muscle cross-sectional area (outlined in yellow), muscle thickness (yellow arrow), and subcutaneous adipose thickness (red arrow) of the rectus femoris. Tracing was performed using ImageJ software.

Leg extensor morphology was assessed with B-mode ultrasonography (Logiq e, GE Healthcare, Chicago, IL) following Herda et al. (24). With participants supine, panoramic transverse images were captured at standardized landmarks: rectus femoris (50% patella to greater trochanter distance), vastus lateralis (40% lateral-epicondyle to anterior superior iliac spine), and vastus medialis (20% medial-epicondyle to anterior superior iliac spine). A custom foam-padded probe guide ensured perpendicular sweeps while minimal pressure and ample gel prevented compression. ImageJ (National Institutes of Health, Bethesda, MD) was used to trace muscle cross-sectional area (mCSA, cm^2^) and extract mean echo intensity (mEI, grayscale score from 0 to 255) from the same region; muscle and subcutaneous-fat thickness were measured with the straight-line tool, as per Cleary et al. (25). Two participants did not complete this assessment. Figure 1 shows a representative rectus femoris image.

#### 2.4.4 Anthropometrics and body composition

Height and weight were measured using a calibrated stadiometer and digital scale. Body mass index (BMI) was calculated as weight (kg) divided by height (m^2^). Waist circumference was measured using a standard tape measure (26). Body composition was assessed using bioelectrical impedance analysis (BIA) (Bodystat Quadscan 4,000) (27). Percent lean body mass was used as the primary measure of body composition.

### 2.5 NMJ integrity measurement

NMJ degeneration is a feature of AD (28) that can be assessed by measuring C-terminal agrin fragment (CAF), a biomarker that may also predict physical function (28). Thus, fasting plasma CAF levels were quantified in duplicate using an enzyme-linked immunosorbent assay (#ab216945, Abcam, Cambridge, United Kingdom). Per manufacturer protocol, 50 μL of 4-fold diluted plasma or standard solution was added to pre-coated wells, followed by 50 μL of the CAF antibody cocktail. The plate was incubated at room temperature on a plate shaker set to 400 rpm for 1 hour. After incubation, the wells were washed three times with 1 × Wash Buffer PT. Next, 100 μL of TMB development solution was added, and the plate was incubated in the dark for 8 minutes at 400 rpm. Following this, 100 μL of stop solution was added, and the plate was shaken for 1 minute to ensure thorough mixing. Absorbance was measured at 450 nm using an MR-9600 Accuris Smartreader 96 (Benchmark Scientific, Sayreville, NJ). CAF concentrations were determined by interpolating the absorbance values from a standard curve and adjusting for the 4-fold dilution factor. CAF analysis was not completed for one participant.

### 2.6 Statistical analysis

The primary objective of this study was to investigate whether 8 weeks of CrM supplementation was associated with improvement in muscle strength, size, and NMJ integrity in patients with AD. Continuous data are expressed as mean ± standard deviations, and categorical data are reported as frequencies and percentages. We used paired sample t-tests to analyze mean changes in all measures from baseline to 8 weeks. We used linear mixed models, including the interaction of time and sex with subject ID as a random effect, to explore sex-based differences in muscle strength (handgrip and leg), muscle ultrasonography, percent lean body mass, and CAF measurements. All statistical analyzes were performed using R software (version 4.1.1; R Foundation, Vienna, Austria). A two-sided *p*-value of less than 0.05 was considered statistically significant.

## 3 Results

Twenty participants diagnosed with dementia due to probable AD (73.1 ± 6.3 years) completed the CABA study. The CrM intervention was well-tolerated with no withdrawals due to adverse events and excellent adherence, with 19 of 20 participants (95%) achieving ≥80% compliance and mean self-reported dose compliance of 90.0% based on daily study partner tracker logs, as detailed here (7). Baseline demographic characteristics are presented in Table 1. All outcomes are presented in Table 2.

**Table 1:** CABA baseline demographic characteristics.

Variable	*n* = 20
Age, years	73.1 ± 6.3^a^
Sex (*n*, %)	
Female	7, 35.0%
Male	13, 65.0%
Race, ethnicity (*n*, %)	
African American, not Hispanic	1, 5.0%
Asian, not Hispanic	1, 5.0%
White, not Hispanic	17, 85.0%
Other, Hispanic	1, 5.0%
Education (*n*, %)	
Completed high school	2, 10.0%
Associate’s	6, 30.0%
Bachelor’s	6, 30.0%
Master’s	2, 10.0%
Doctorate, professional	4, 20.0%
Stanford brief physical activity survey	
Inactive	2, 10%
Light	10, 50%
Moderate	8, 40%
Mini-Mental state exam	21.7 ± 4.4

Values are mean ± SD unless indicated as frequency (%).

**Table 2:** Effects of 8 weeks of CrM supplementation on muscle strength, muscle ultrasonography, neuromuscular junction integrity, and body composition.

Variable	Baseline	8 weeks	*p*-value
Muscle strength measures^a^			
Handgrip strength (kg)	33.5 ± 11.6	35.4 ± 11.5	0.02
Peak torque 60 degrees per second^b^ (Nm)	83.7 ± 40.8^c^	84.3 ± 43.2	0.80
Peak torque 120 degrees per second^b^ (Nm)	58.9 ± 31.7	60.5 ± 30.2	0.73
Peak torque 180 degrees per second^b^ (Nm)	41.7 ± 26.7	42.3 ± 22.2	0.90
Ultrasonography measures^d^			
Rectus femoris mCSA (cm^2^)	7.6 ± 2.5	7.8 ± 2.7	0.03
Vastus medialis mCSA (cm^2^)	10.1 ± 3.0	10.2 ± 3.1	0.01
Rectus femoris muscle thickness (cm)	1.6 ± 0.4	1.6 ± 0.4	0.41
Vastus medialis muscle thickness (cm)	1.9 ± 0.3	1.9 ± 0.3	0.37
Vastus lateralis muscle thickness (cm)	1.5 ± 0.3	1.5 ± 0.4	0.17
Rectus femoris mEI	92.2 ± 33.0	99.0 ± 31.4	0.33
Vastus medialis mEI	86.2 ± 25.8	95.3 ± 28.3	0.22
Rectus femoris region subcutaneous fat (cm)	1.09 ± 0.6	1.05 ± 0.6	0.006
Vastus medialis region subcutaneous fat (cm)	0.92 ± 0.5	0.91 ± 0.5	0.52
Vastus lateralis region subcutaneous fat (cm)	0.99 ± 0.6	0.95 ± 0.5	0.003
NMJ integrity^e^			
Plasma CAF (ng/mL)	2.5 ± 0.6	2.6 ± 0.8	0.46
Anthropometric and body composition measures			
BMI (kg/m^2^)	25.4 ± 3.7	25.2 ± 3.5	0.25
Percent lean body mass^e^	69.1 ± 7.9	71.4 ± 8.5	0.10
Waist circumference (cm)	93.3 ± 9.1	92.1 ± 9.0	0.20

a Mean Baseline and 8 weeks differences were assessed using a paired samples *t*-test. Significance was set at *p* < 0.05.

b *n* = 10.

c Mean ± SD – all such values.

d *n* = 18.

e *n* = 19.

CrM, creatine monohydrate; NMJ, neuromuscular junction; Nm, Newton-meter; mCSA, muscle cross-sectional area; mEI, muscle echo intensity; CAF, C-terminal agrin fragment; BMI, body mass index.

### 3.1 Muscle strength

**Figure 2:**
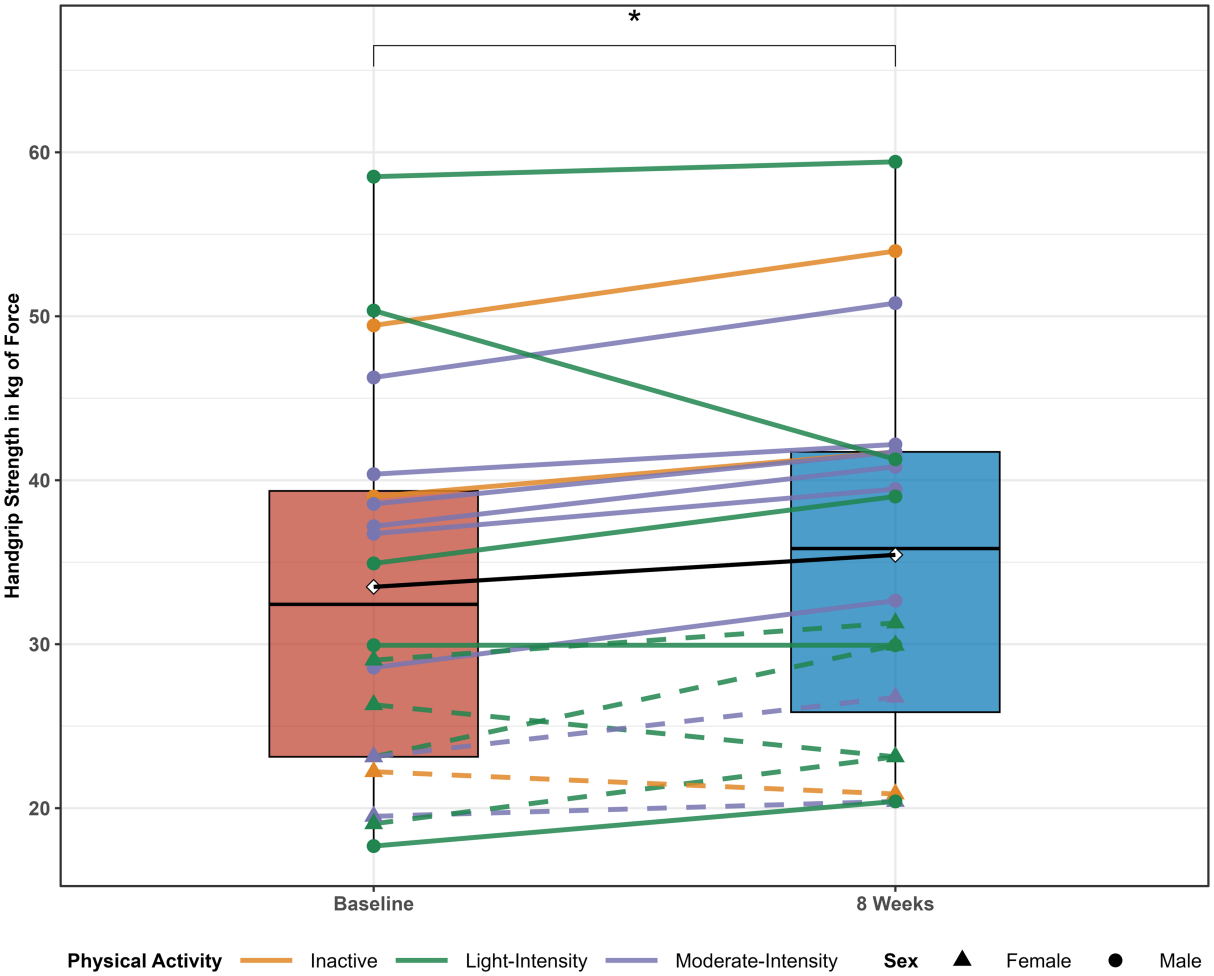
Time point comparisons and individual changes in handgrip strength after 8 weeks of creatine monohydrate supplementation. Boxplots display strength values at baseline and after 8 weeks, with individual trajectories overlaid. Line color reflects baseline physical activity level: orange for inactive, green for light-intensity, and purple for moderate-intensity. Solid lines with circular markers represent male participants, while dashed lines with triangular markers represent female participants. * *p* < 0.05.

Handgrip strength increased from baseline to 8 weeks (33.5 ± 11.6 kg vs. 35.4 ± 11.5 kg, *p* = 0.02). Figure 2 illustrates mean changes in handgrip strength from baseline to 8 weeks.

Due to mechanical issues with the leg dynamometer, only ten CABA participants completed leg dynamometry to measure leg strength. Peak torque did not change for any of the three velocities tested: (1.05 rad∙s^−1^: 83.7 ± 40.8 Nm vs. 84.3 ± 43.2 Nm, *p* = 0.80; 2.10 rad∙s^−1^: 58.9 ± 31.7 Nm vs. 60.5 ± 30.2 Nm, *p* = 0.73; 3.14 rad∙s^−1^: 41.7 ± 26.7 Nm vs. 42.3 ± 22.2 Nm, *p* = 0.90). There were no differences by sex for changes in either hand or leg strength, despite males having higher baseline strength than females.

### 3.2 Leg extensor ultrasonography

Eighteen CABA participants underwent ultrasonography to measure leg muscle size at baseline and 8 weeks. mCSA increased in the rectus femoris (7.6 ± 2.5 cm^2^ vs. 7.8 ± 2.7 cm^2^, *p* = 0.03) and vastus medialis (10.1 ± 3.0 cm^2^ vs. 10.2 ± 3.1 cm^2^, *p* = 0.01).

Muscle thickness did not change for any muscles: rectus femoris (1.6 ± 0.4 cm vs. 1.6 ± 0.4 cm, *p* = 0.41; vastus medialis 1.9 ± 0.3 cm vs. 1.9 ± 0.3 cm, *p* = 0.37; and vastus lateralis 1.5 ± 0.3 cm vs. 1.5 ± 0.4 cm, *p* = 0.17). Similarly, no significant changes were observed in mEI (rectus femoris: 92.2 ± 33.0 cm vs. 99.0 ± 31.4 cm, *p* = 0.33; vastus medialis: 86.2 ± 25.8 cm vs. 95.3 ± 28.3 cm, *p* = 0.22). In contrast, subcutaneous fat decreased in the rectus femoris region (1.09 ± 0.6 cm vs. 1.05 ± 0.6 cm, *p* = 0.006) and vastus lateralis region (0.99 ± 0.6 cm vs. 0.95 ± 0.5 cm, *p* = 0.003). No changes were observed in the vastus medialis region (0.92 ± 0.5 cm vs. 0.91 ± 0.5 cm, *p* = 0.52). There were no differences by sex for changes in all ultrasonography measures, despite males having larger mCSA in the rectus femoris and vastus medialis than females.

### 3.3 Anthropometrics and body composition

Twenty participants completed BMI and waist circumference measurements at both baseline and the 8-week visit, while 19 completed BIA. BMI did not change from baseline to 8 weeks (25.4 ± 3.7 kg/m^2^ vs. 25.2 ± 3.5 kg/m^2^, *p* = 0.25). Similarly, percent lean body mass showed no significant change (69.1 ± 7.9% vs. 71.4 ± 8.5%, *p* = 0.10), nor did waist circumference (93.3 ± 9.1 cm vs. 92.1 ± 9.0 cm, *p* = 0.20). There were no differences by sex for changes in percent lean body mass, despite males having greater percent lean body mass than females.

### 3.4 NMJ integrity

Nineteen participants completed CAF measurements at both baseline and the 8-week visit. Plasma CAF concentrations did not change form baseline to 8 weeks (2.5 ± 0.6 ng/mL vs. 2.6 ± 0.8 ng/mL, *p* = 0.46). There were no differences by sex for changes in CAF.

## 4 Discussion

Results from this pilot study suggest 8 weeks of CrM supplementation is associated with modest improvement in muscle strength and size in patients with AD. This is the first study to test whether CrM may benefit skeletal muscle in AD, and these preliminary gains justify larger, controlled trials to investigate its promise as a low-cost strategy for slowing AD-related decline in muscle health.

We observed associated improvements in upper body muscle strength following 8 weeks of CrM supplementation, with a 1.9 kg (~6%) increase in handgrip strength, a reliable proxy of upper body strength. This improvement is clinically meaningful as handgrip strength is associated with quality of life in older adults (29) and mortality in patients with AD (30). While we are the first to test CrM supplementation in patients with AD, similar handgrip strength improvements have been documented in healthy older adults (10). Although our leg strength data were limited to a subset of participants (*n* = 10), we did not observe statistically significant changes in leg strength. These results should be interpreted with considerable caution due to the lack of standardized familiarization trials, which are essential for reliable strength measurements in older adults and may have resulted in underestimation of true strength or learning effects (24). While maintaining leg strength over 8 weeks may be clinically important in AD, where progressive muscle weakness is common (1), the methodological limitations prevent us from drawing definitive conclusions about CrM’s effects on lower extremity strength. Nevertheless, the feasibility of conducting isokinetic dynamometry in this population was demonstrated, suggesting potential utility for future studies with proper methodological controls. Together, the improvement in handgrip strength aligns with previous studies showing CrM benefits in other populations and suggest that CrM may help preserve overall muscle function in AD. Future studies should incorporate standardized familiarization protocols and consider combining CrM supplementation with resistance training or cognitive-motor interventions, as this approach improves muscle outcomes more than supplementation alone (8–10, 31) and may be particularly beneficial for optimizing muscle strength and function in patients with AD.

We observed modest but statistically significant associated improvements in mCSA in the rectus femoris (+0.2 cm^2^) and vastus medialis (+0.1 cm^2^), muscle groups where larger mCSA are generally associated with greater leg strength (32). However, we did not observe corresponding leg strength improvements in our limited sample. Our findings align with well-documented benefits of CrM for muscle size in other populations (8, 9, 11–13). In addition to these changes in mCSA, we also noted a localized decrease in subcutaneous fat thickness in the rectus femoris and vastus lateralis, despite stable overall body weight and composition. This localized decrease in subcutaneous fat thickness, occurring alongside increases in muscle size, suggests that CrM supplementation may promote favorable changes in muscle-to-fat ratio at the tissue level, independent of overall body composition changes. Although the mCSA increases were small, they may still be clinically relevant as they represent preservation/gains that could help offset the 1–2% annual age-related muscle loss typically seen in older adults (33), which may be accelerated in AD (1). Modest gains in muscle size can improve functional capacity (34) and glucose metabolism (35), which is perturbed in AD (36). Moreover, since mCSA captures only a portion of total muscle volume, a 0.2 cm^2^ increase could reflect a more substantial hypertrophic response. While we cannot rule out that some mCSA increase is due to CrM-related muscle hydration (37), the concurrent improvement in handgrip strength supports that the increases in mCSA may have functional implications. Taken together, these findings, though based on a brief, eight-week trial, suggest that CrM supplementation may help preserve muscle size and support functional improvements in individuals with AD, a population particularly vulnerable to muscle loss (38–40).

We assessed NMJ integrity using CAF as a biomarker of NMJ degeneration but found no statistically significant changes after 8 weeks of CrM supplementation. This lack of change may reflect that the eight-week intervention duration was too short to observe meaningful changes in NMJ biomarker; NMJ remodeling and regeneration are complex processes that may require months rather than weeks to manifest detectable changes in circulating biomarkers (41). Our patients also showed relatively preserved NMJ integrity compared to other AD cohorts (28) and age-matched healthy controls (42), which may have limited the room for improvement. Additionally, CAF may have inherent variability and sensitivity limitations (43), and CAF levels may be influenced by factors beyond creatine supplementation, such as physical activity, inflammation, or disease progression rates, which could mask potential treatment effects in a heterogeneous AD population. Investigating CrM’s effects on NMJ integrity may require longer intervention periods, larger sample sizes, and more sensitive biomarkers or complementary assessments such as electromyography to capture subtle neuromuscular changes that circulating biomarkers might miss.

Although our study did not directly investigate mechanisms, several pathways may explain CrM’s association with improved muscle outcomes. The ATP deficits documented in both skeletal muscle (44) and neurons (36) in AD patients may make this population especially responsive to Cr′s bioenergetic support. Beyond the basic energy metabolism pathways, the concurrent improvements in both muscle size and strength in study suggest activation of anabolic signaling by upregulating muscle protein synthesis (45), downregulating growth-inhibiting proteins such as myostatin (46), and enhancing anabolic signaling through the mammalian target of rapamycin pathway (47) and insulin-like growth factor (48). The antioxidant properties of Cr (49, 50) may be particularly beneficial in AD, where oxidative stress drives both muscle deterioration and neurodegeneration (51, 52), potentially explaining the muscle preservation we observed. Together, these mechanisms may underlie the gains in muscle strength and size observed in this study, though mechanistic studies are needed to determine which pathways are most relevant to CrM’s effects in AD populations.

Our pilot trial suggests that CrM may offer valuable benefits for AD-related functional decline in patients with AD; however, as a single-arm pilot study with limited racial and sex diversity and designed to generate preliminary data rather than provide definitive evidence, these findings should be interpreted cautiously. The lack of a control group, small sample size, and short eight-week duration all limit the strength of our conclusions. Additionally, our leg strength assessment was limited by mechanical issues with the dynamometer, requiring mid-study protocol changes that reduced our sample size and prevented standardized familiarization trials, which are essential for reliable leg strength data in older adults (24). Finally, ultrasonography assessments were conducted without standardized participant hydration protocols, which can affect measurements of mCSA and muscle thickness, and without assessor blinding to participant identity and time point, potentially introducing measurement bias. Future randomized, placebo-controlled trials with larger sample sizes, longer intervention durations of 12–24 weeks, and standardized protocols are needed to confirm these preliminary findings and capture more robust muscle and neuromuscular adaptations.

## 5 Conclusion

Although our study is limited by its single-arm nature, our study provides preliminary evidence that CrM supplementation is associated with improvements in upper body strength and lower body muscle size in patients with AD. Enhancing skeletal muscle strength and size may help prevent AD-related decline in physical function, potentially slowing functional decline and improving quality of life. As a cost-effective, well-tolerated intervention, CrM represents a promising adjuvant therapeutic strategy that warrants investigation in larger randomized controlled trials to establish its efficacy for preserving physical function in patients with AD.

## Data Availability

The raw data supporting the conclusions of this article will be made available by the authors, without undue reservation.
